# Amontillado is required for *Drosophila* Slit processing and for tendon-mediated muscle patterning

**DOI:** 10.1242/bio.020636

**Published:** 2016-09-14

**Authors:** Elly Ordan, Talila Volk

**Affiliations:** Department of Molecular Genetics, Weizmann Institute of Science, Rehovot 76100, Israel

**Keywords:** Amontillado, *Drosophila*, Embryo, Muscle, Slit cleavage, Tendon

## Abstract

Slit cleavage into N-terminal and C-terminal polypeptides is essential for restricting the range of Slit activity. Although the Slit cleavage site has been characterized previously and is evolutionally conserved, the identity of the protease that cleaves Slit remains elusive. Our previous analysis indicated that Slit cleavage is essential to immobilize the active Slit-N at the tendon cell surfaces, mediating the arrest of muscle elongation. In an attempt to identify the protease required for Slit cleavage we performed an RNAi-based assay in the ectoderm and followed the process of elongation of the lateral transverse muscles toward tendon cells. The screen led to the identification of the *Drosophila* homolog of pheromone convertase 2 (PC2), Amontillado (Amon), as an essential protease for Slit cleavage. Further analysis indicated that Slit mobility on SDS polyacrylamide gel electrophoresis (SDS-PAGE) is slightly up-shifted in *amon* mutants, and its conventional cleavage into the Slit-N and Slit-C polypeptides is attenuated. Consistent with the requirement for amon to promote Slit cleavage and membrane immobilization of Slit-N, the muscle phenotype of *amon* mutant embryos was rescued by co-expressing a membrane-bound form of full-length Slit lacking the cleavage site and knocked into the *slit* locus. The identification of a novel protease component essential for Slit processing may represent an additional regulatory step in the Slit signaling pathway.

## Abstract (precis)

**Summary:** The *Drosophila* homolog of pheromone convertase 2 (PC2), amontillado (Amon), is shown to contribute to Slit processing and further cleavage into an N-Slit, essential for Slit activity in directing muscle patterning.

## INTRODUCTION

A functional musculoskeletal system depends on proper recognition and connection of muscles and tendons (Schweitzer et al., 2010). In the *Drosophila* embryo each muscle is formed through fusion of a single founder cell with fusion-competent myoblasts, forming a syncytium that then elongates towards its attachment sites, the ectodermal tendon cells (Schejter and Baylies, 2010; Schnorrer and Dickson, 2004b; Soler et al., 2016). Once the elongating muscle comes in contact with its corresponding tendon, an integrin-mediated myotendinous junction (MTJ) is formed between both cell types (Bokel and Brown, 2002). In the embryo, each of the abdominal hemi-segments contains thirty different types of muscles that are all formed and elongate within a short window of time (Bate, 1990).

An important question in the field is how the different muscles find the correct path and attach to the right tendon. Based on a growing amount of data, it has been suggested that different muscle types exhibit an individual intrinsic polarity, independent of tendon signals, that governs their basic directionality (Krzemien et al., 2012; Ordan et al., 2015; Schnorrer and Dickson, 2004a). The external signals provided by tendons are of a short-range nature, and are necessary for fine-tuning the muscle path, either by repulsion or by providing a stop signal at the presumptive attachment site (Ordan and Volk, 2015a). An essential signal for directing muscle elongation is mediated by the secreted protein Slit. Slit function is best characterized in the central nervous system (CNS) where the midline glia secrete Slit, thus repelling neurons by binding to the Slit receptor/s Robo1, 2, and 3 (Chedotal, 2007; Dickson and Gilestro, 2006; Fan et al., 2003; Kidd et al., 1999). Muscle cells have been shown to express Robo, whereas tendons express Slit (Kramer et al., 2001). Slit is a large protein with distinct functional domains, including four leucine-rich repeats (LRR), nine epidermal growth factor (EGF) repeats, and a laminin-G domain, as well as a highly conserved proteolytic cleavage site (Chedotal, 2007). Slit cleavage leads to the formation of two polypeptides: a ∼90 kDa N-terminal (Slit-N) that contains the Robo binding site, and a ∼80 kDa C-terminal fragment (Slit-C) (Brose et al., 1999; Wang et al., 1999). Full-length Slit and Slit-N were shown to be more tightly associated with the cell surface and Slit-C is mostly shed into extracellular space (Brose et al., 1999). Both Slit-FL and Slit-N bind Robo receptors induce cellular repulsion (Nguyen Ba-Charvet et al., 2001; Wang et al., 1999). Our previous analysis demonstrated that Slit cleavage is essential for proper elongation of the lateral transverse muscles 1-3 (LT1-3), as well as for the extension of the dorsal acute muscle 3 (DA3) towards its corresponding tendon attachment cell. Moreover, these results indicated that, upon cleavage, Slit-N remains tethered to the cell surfaces of the tendon cell, forming a short-range repulsive signal that is important for keeping the elongating LT muscles on their correct paths in the middle of the segment, and also for providing a stop signal for the DA3 muscle once it has reached the tendon site (Ordan et al., 2015). The cleavage of Slit is tightly controlled at the tendon site and a non-signaling role of Robo2 expressed by the tendon cells is required to support this cleavage, presumably by cis-association with Slit (Ordan and Volk, 2015b). Additional studies further argue for distinct activities exhibited by Slit-N or Slit-C in neurons in vertebrates, as well as in non-neuronal cells (Coleman et al., 2010; Dascenco et al., 2015; Shrivastava et al., 2015; Svensson et al., 2016; Wright et al., 2012), emphasizing a functional regulatory significance for Slit cleavage. However, despite the identification of Slit cleavage site, the protease that cleaves Slit has not yet been identified.

In an attempt to identify this protease we used a candidate-based RNAi screen for secreted or membrane-bound proteases in the *Drosophila* genome that, when knocked down in the ectoderm, impaired muscle pattern. We have identified *amontillado* (*amon*) as an essential gene for muscle patterning. Amon is the *Drosophila* homolog of pheromone convertase 2 (PC2) (Hwang et al., 2000; Rayburn et al., 2003; Rhea et al., 2010; Siekhaus and Fuller, 1999). It belongs to a family of serine proteases, the subtilisin-like proprotein convertases (SPCs) that typically cleave precursor proteins after single or paired basic residues. PC2 displays a neuroendocrine tissue-specific expression pattern (Smeekens et al., 1991) and is responsible for cleavage and activation of peptide hormones. In *Drosophila*, Amon has been shown to be essential for locomotion, molting, and pupal development. During embryogenesis, *amon* mutants are semi-lethal and exhibit reduced movement. Amon's role in molting can be rescued by a burst of expression in a subset of neurons. Our present findings imply an essential role for Amon in muscle patterning during embryogenesis and further show that Amon is necessary for Slit cleavage. Consistent with the contribution from Amon to Slit cleavage, the muscle phenotype of *amon* mutants can be rescued by membrane tethered cleaved Slit-N. These results suggest that Amon mediates Slit cleavage in the embryo.

## RESULTS AND DISCUSSION

### Amon is required for Slit-dependent muscle elongation

**Fig. 1.**
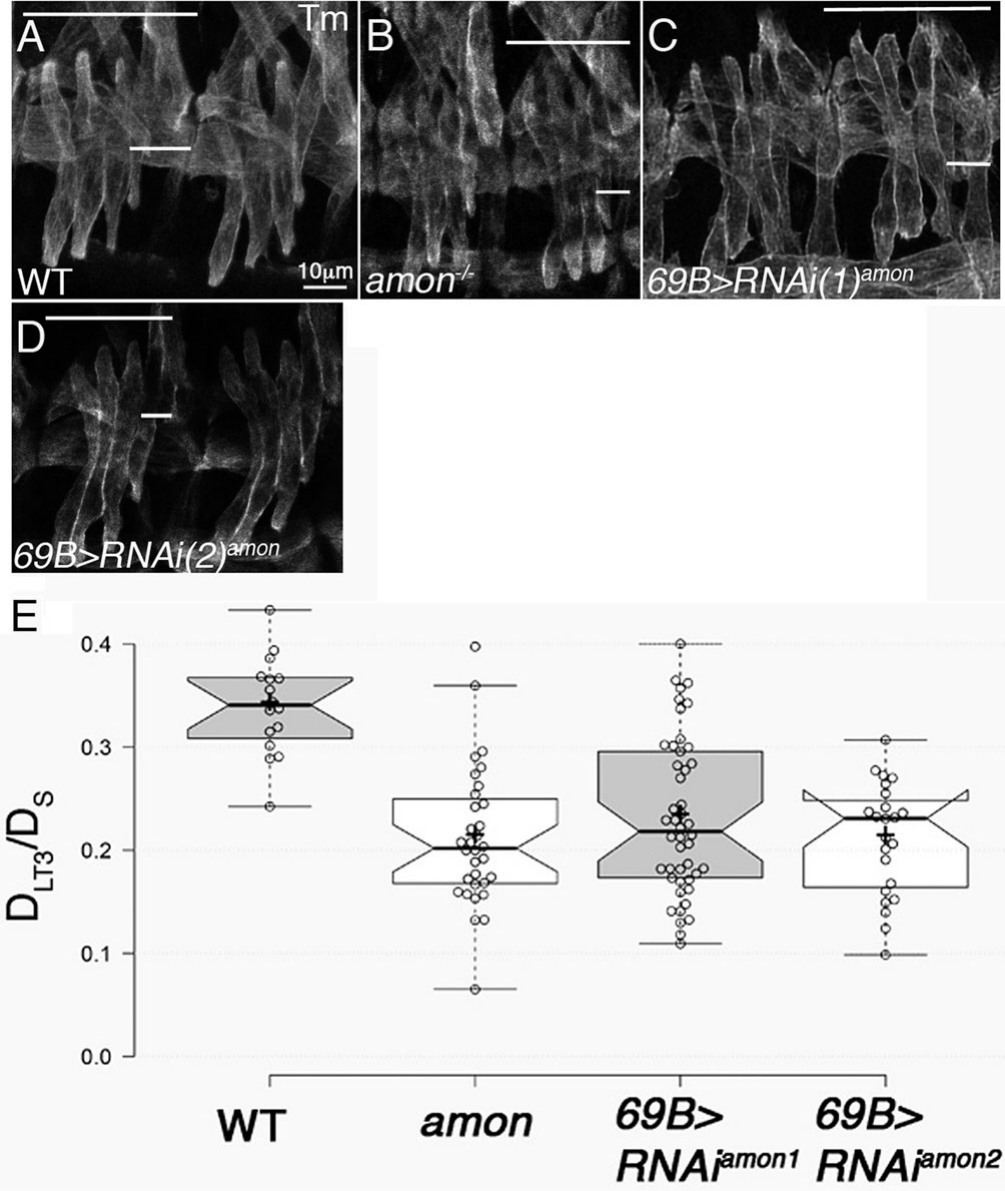
**Amon is essential for proper muscle patterning.** The lateral transverse (LT) muscles in embryos expressing two distinct RNAi against Amon driven by the whole ectoderm driver *69b-Gal4* are closer to the segmental border (compare C,D to A). A similar phenotype is observed in *amon* homozygous mutant embryos (B). All muscles are labeled with anti Tropomyosin (Tm). In all images the long horizontal line represents segment width (D_s_), and the short line represents the distance of LT3 muscle from the posterior border (D_L3_). Bar in A represents 10µm and is equal in all panels. (E) Quantification of the distance between LT3 muscle and the segment border (D_L3_) divided by the entire segmental length (D_s_) in WT, *amon*, 69B-Gal4<RNAi^amon1^, and 69B-Gal4<RNAi^amon2^. Center lines show the medians; box limits indicate the 25th and 75th percentiles as determined by R software; whiskers extend 1.5 times the interquartile range from the 25th and 75th percentiles, outliers are represented by dots; crosses represent sample means; bars indicate 83% confidence intervals of the means; data points are plotted as open circles. Gray and white represent the different experiments.

In order to identify the protease that cleaves Slit, an RNAi-based assay of candidate extracellular proteases, whose knock-down in the ectoderm leads to a Slit-like muscle phenotype, was conducted. The screen was performed using the ectodermal driver *69B-Gal4*, which drives expression before muscle elongation in the entire ectoderm. The genes tested are summarized in Table 1 and include extracellular or membrane-tethered proteases that share signal peptide and/or transmembrane domain, and in addition, a well-characterized, evolutionary conserved protease domain. Out of five candidates, one (*amon*) exhibited a muscle phenotype similar to that of *slit* mutants when knocked down in the ectoderm. In embryos expressing *amon* RNAi in the ectoderm the LT muscles were closer to the segmental border (Fig. 1C,D), similar to the phenotype observed in *slit* mutants (Ordan et al., 2015), *robo,robo3* mutants (Ordan and Volk, 2015a,b), and homozygous *amon* mutant embryos (Fig. 1B). For quantitation of the LT muscle phenotype, the ratio between the distance of muscle LT3 to the posterior segmental border (D_LT3_) and the width of the entire segment (D_S_) was calculated in segments of the mutant embryos (Fig. 1E), as was previously performed for *slit* mutants. This method was also validated by calculating the ratio of the entire area between the LT3 muscle and the segment border to the entire segment area in both RNAi knock-down embryos (using two different RNAi lines), as well as in the homozygous *amon* mutants. The ratio of D_LT3_/D_S_ was 57% smaller than in wild type, implying that the LT muscles were significantly closer to the segmental border compared with wild-type embryos (*P*=0.001). Importantly, a 60% reduction in the ratio of D_LT3_/D_S_ was previously reported in *slit* mutants (Ordan et al., 2015), and a 61% reduction in that ratio was reported in *robo;robo3* mutant muscles (Ordan and Volk, 2015a,b). These results are consistent with the involvement of Amon protease in Slit processing.

**Table 1.**
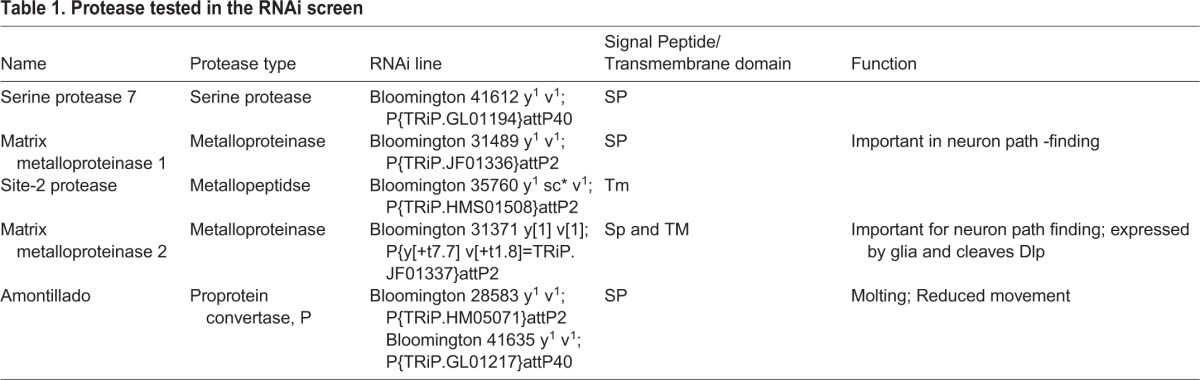
**Protease tested in the RNAi screen**

### Amon is essential for slit cleavage

**Fig. 2.**
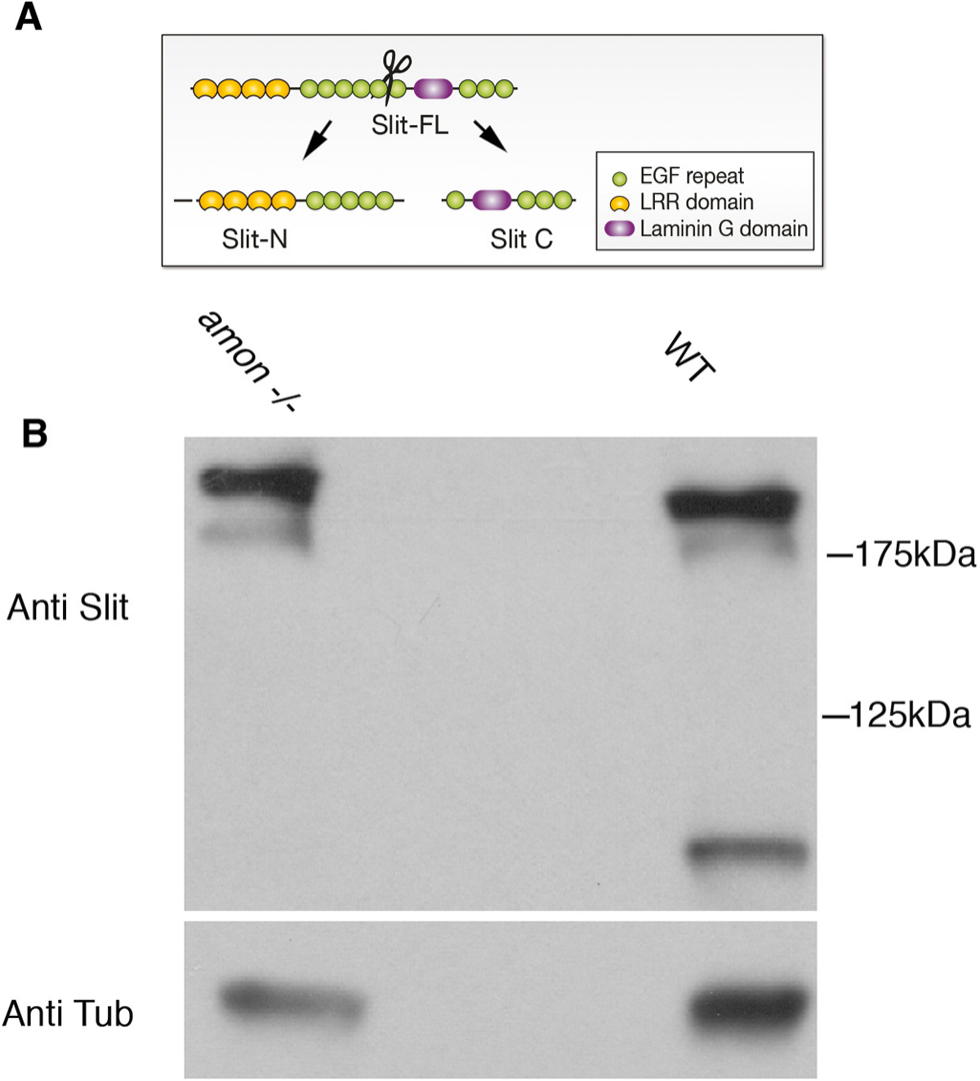
**Amon is essential for Slit cleavage.** (A) A scheme of Slit-cleaved polypeptides is presented. (B) Western blot analysis with anti-Slit protein extract taken from stage 16 embryos, of WT (right lane) and homozygous *amon* mutants (left lane). A ∼180 kDa high molecular weight bands representing full length Slit are detected in both WT and *amon* mutants. The cleaved ∼80 kDa Slit C-terminal band is detected in WT but not in *amon* mutants. The same blot was reacted with anti-Tub showing a comparable amount of αTub in both extracts. These results are representative of 3 similar, independent analyses.

In order to test whether Amon is important for Slit cleavage, a western blot with anti-Slit antibody of stage 16 embryos of wild-type (WT) and *amon* homozygous mutants was performed. Mutant embryos were identified by using a GFP balancer and selected under a fluorescent binocular. In extracts from WT embryos, two bands are observed by the antibody: a high molecular weight band of about 180 kDa representing the full length Slit, and a lower molecular weight band of ∼80 kDa representing the cleaved Slit-C polypeptide. The antibody does not recognize the Slit-N polypeptide. In wild-type embryos full length Slit and Slit-C are clearly observed. In contrast, in *amon* homozygous mutants the full length Slit is detected whereas a very faint band of Slit-C is detected (Fig. 2). Interestingly, the band of full length Slit appears slightly higher than the wild-type Slit, implicating a role for Amon in processing of Slit, reducing its size to the ∼180 kDa band that is normally detected. These results demonstrate that Slit cleavage is significantly reduced in *amon* mutants, possibly due to the lack of its processing by Amon.

**Fig. 3.**
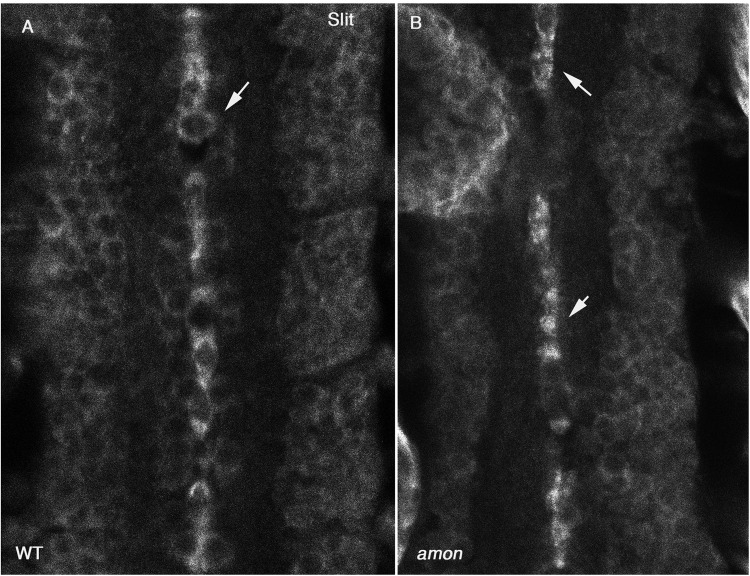
**Slit accumulates in the cytoplasm of midline glia cells in *amon* mutants.** Staining of anti Slit antibody marks the midline glial surfaces in wild-type embryos (arrow in A), whereas in *amon* mutant midline glia it shows intracellular dots (arrows in B).

Previous analysis indicated that loss of *amon* correlated with loss of neuropeptide hormone signals from the larval ring gland and perisympathetic organs (Wegener et al., 2011). These peptides undergo multiple intra- and extra-cellular cleavages, and Amon was predicted to cleave intracellularly in the trans-Golgi compartment. An additional report demonstrated the requirements of Amon for processing of the glucose regulatory hormone AKH, leading to reduced hemolymph sugar levels (Rhea et al., 2010). It appears, therefore, that Amon might function in distinct cell types and be required for pleiotropic processes. Whereas the processing of AKH might not be direct, it clearly initiates a cascade of proteolytic cleavages, which eventually leads to the lack of AKH, or other neuropeptides. This scenario might also be relevant to the involvement of Amon in Slit processing. In an attempt to prove a direct link between Amon and Slit, we co-expressed Amon in S2 cells together with Slit and 7B2 helper protein, however, we were not able to detect enhanced Slit cleavage. A possible explanation is that both Slit and Amon are secreted into the medium, preventing their efficient encounter. Likewise, overexpression of Amon in the embryonic ectoderm did not lead to enhanced Slit cleavage. It was therefore difficult to determine whether Amon directly cleaves Slit, or initiates a proteinase cascade at the end of which Slit is cleaved. Interestingly, the molecular weight of Slit in *amon* mutant embryos was slightly higher than that in wild-type embryos, consistent with the involvement of Amon in processing full-length Slit into a slightly smaller polypeptide. Importantly, unlike its surface distribution in the midline glia of wild-type embryos (stage 16), Slit appears to accumulate in a dot-like pattern within the cytoplasm of *amon* mutant embryos (Fig. 3A,B), implicating that Amon is essential for its intracellular processing for proper secretion. Both the differential size of Slit, and its abnormal cytoplasmic accumulation, are consistent with a role for Amon in cytoplasmic Slit processing essential for proper secretion and cleavage into N-Slit.

### Expression of membrane-bound uncleavable Slit bypasses the requirement for Amon

**Fig. 4.**
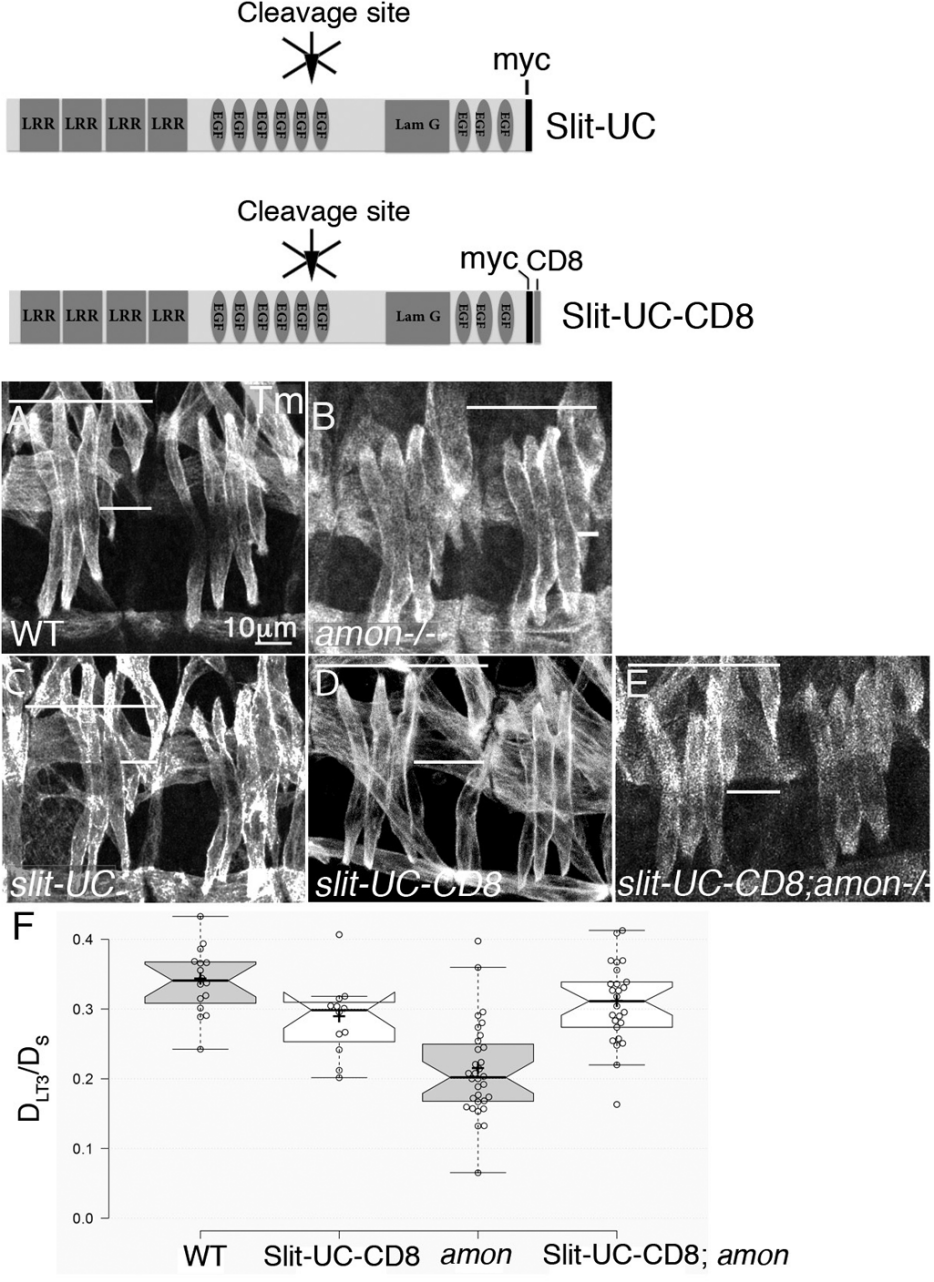
**Membrane-bound uncleavable Slit rescues the pattern of LT muscles in *amon* mutants.** Upper panel indicates the knocked-in slit constructs. (A-E) Representative images of the LT muscles in WT (A), *amon* (B), knocked-in uncleavable slit (C), knocked-in uncleavable slit with CD8 (D), and a recombinant carrying knocked-in uncleavable Slit with *amon* mutant allele (E). All muscles are labeled with anti Tropomyosin (Tm). Bar in A represents 10 µm, indicating the magnification in all panels. (F) Quantification of the ration D_LT3_/D_S_, indicating a significant difference between *slit-UC-CD8;amon* and *amon* alone. Center lines show the medians; box limits indicate the 25th and 75th percentiles as determined by R software; whiskers extend 1.5 times the interquartile range from the 25th and 75th percentiles, outliers are represented by dots; crosses represent sample means; bars indicate 83% confidence intervals of the means; data points are plotted as open circles. Gray and white represent the different experiments.

Our previous analysis demonstrated that Slit cleavage is essential to allow anchoring of Slit-N to the tendon cell surfaces (Ordan et al., 2015). Association of Slit-N with the tendon cell membrane restricts its range of activity, only affecting the approaching muscle. Therefore, circumventing the need for Slit cleavage can be achieved by anchoring an uncleavable form of full-length Slit to the membrane (Ordan et al., 2015) (Fig. 4D). If the requirement for Amon in the process of muscle elongation is based on its activity in Slit processing, it was predicted that replacing full-length Slit with uncleavable Slit, which also has a membrane-bound domain (Slit-UC-CD8), would rescue the muscle phenotype of *amon.* To this end, Slit-UC-CD8 knocked-in to the Slit locus was combined with an *amon* mutant allele, and elongation of the LT muscles in homozygous *amon* mutant embryos and knocked-in Slit-UC-CD8 was analyzed. The path of these muscles was rescued in comparison to homozygous *amon* mutants alone (*P*=0.0001). Representative images and quantification of the elongation of the LT muscles in *amon;slit-UC-CD8* in comparison to the control of WT, *amon*, *slit-UC*, and *slit-UC-CD8* alone are shown in Fig. 4. These experiments indicated that proper elongation of the LT muscles depends on Amon-induced Slit processing into a membrane-bound polypeptide.

In summary, the PC2 protease family member Amon is shown to promote Slit cleavage in the *Drosophila* embryo. Its reported expression and activity in the nervous system is consistent with its ability to promote Slit processing in this system. Because knockdown of Amon in the ectoderm led to a muscle phenotype, it is assumed that Amon is expressed by the ectoderm, including the tendon cells, where it promotes Slit processing inside these cells, an activity that is required for further Slit cleavage to Slit-N and Slit-C polypeptides.

## MATERIALS AND METHODS

### Fly lines

*yw*, *69b-gal4*, Amon RNAi 1 and 2 (Bloomington stock no. 41635 and 28583), Sp7 RNAi (Bloomington stock no. 41612), MMP1 RNAi (Bloomington stock no. 31489), MMP2 RNAi (Bloomington stock no. 31371), *amon* mutant (Bloomington stock no. 29011) and *slit2* were received from the Bloomington Stock Center. Slit knock-in lines were described in Ordan et al. (2015) and produced in the Dickson lab.

### Antibodies

Antibodies used rat anti-Tropomyosin (1:400; Abcam, Ab-50567), mouse anti-Slit (1:1000; Developmental Studies Hybridoma Bank, The University of Iowa). Secondary fluorescent antibodies were purchased from Jackson Laboratories.

### Immunostaining

Staged embryos were collected and fixed as previously described (Ashburner et al., 2005). Embryos were visualized with a Zeiss LSM710/LSM780 confocal system. Images were deconvoluted using AutoQuant (Media Cybernetics, AutoQuant X3) and processed using Adobe Photoshop.

### Western blot

Protein extract was produced from staged embryos in RIPA buffer (10mM Tris/HCL pH=7.5, 150mM NaCl, 0.5mM EDTA, 0.5% NP-40). Extract was boiled in sample buffer and run on a 7% SDS-PAGE gel, blotted onto nitrocellulose and reacted with mouse anti-Slit antibody (1:1000; Developmental Studies Hybridoma Bank) and HRP-conjugated anti-mouse IgG (1:5000; Jackson ImmunoResearch, 715-035-151). The signal was visualized using SuperSignal (Thermo Scientific).

### Measurements

Distances were measured using the line tool in ImageJ. A minimum of 5 embryos per genotype were used. Significance was calculated by *t*-test. Box plots were calculated according to Spitzer et al. (2014).
